# Loneliness, health and applied psychology

**DOI:** 10.1111/aphw.12417

**Published:** 2022-11-20

**Authors:** Andrew Steptoe

**Affiliations:** ^1^ Department of Behavioural Science and Health University College London London UK

**Keywords:** Covid‐19, intervention, policy, social isolation

## INTRODUCTION

The impact of loneliness on health is receiving increasing attention throughout the world not only from the research community but also from policy makers, governments and voluntary sector organisations. There have been large‐scale reviews over recent years from organisations such as the National Academy of Sciences, Engineering and Medicine in the USA (2020) and the EU Science Hub (Baarck & Kaovacic, 2022). These initiatives have built on scientific research that is now moving beyond Western high income countries across the world, a trend that has received additional impetus from the COVID‐19 pandemic (Holt‐Lunstad, 2022; Kola et al., 2021; Surkalim et al., 2022). At the policy level, the UK has developed a national strategy on loneliness, appointing a Minister for Loneliness and a cross‐governmental programme of initiatives to reduce loneliness (Department for Digital, Culture, Media, & Sport, 2022). Japan has also created a Cabinet post for a Minister of Loneliness to oversee a programme to support people facing loneliness, stimulated in part by the disturbing rates of suicide in the country. Coordinated initiatives to address loneliness across the life course are being developed in multiple European countries as well and form an important part of ageing strategies (Sandu et al., 2021; Sowa‐Kofta et al., 2021).

Applied psychology is fundamental to these developments and provides much of the intellectual and empirical foundation of loneliness research. In keeping with this international perspective, it is very welcome that the authors contributing to this special issue of *Applied Psychology ‐ Health and Well‐Being* come from across the world: Germany, the UK, China, Japan, Singapore, the United States and Canada. This high‐quality research uses a range of methods, including experimental studies, quantitative and qualitative analyses, meta‐analysis, epidemiological and mechanistic approaches. The articles are focused on three broad issues in loneliness and health research: the relevance of loneliness to the development and maintenance of mental and physical ill‐health, the mechanisms underlying links between loneliness and poor mental health and interventions aimed at reducing loneliness and improving health and well‐being. Several of the contributions focus specifically on the COVID‐19 pandemic when restrictions in socialising and going out of the home, and the closure in many countries of work places and schools, led to particular challenges to social connectedness.

Space prohibits a detailed appraisal of each of these contributions. My comments therefore draw out a set of major themes that are illustrated by this collection and endeavour to place these in the broader context of applied psychological research on mental and physical health.

## LONELINESS IN THE DEVELOPMENT AND MAINTENANCE OF ILL‐HEALTH

A substantial body of longitudinal population research has focused on establishing the role of loneliness in the development and maintenance of ill‐health (Holt‐Lunstad et al., 2015). These studies typically involve measuring loneliness at baseline, then following people up for future health problems or death. This work is primarily designed to understand the links between loneliness and health outcomes, but commentators have also used it to enhance the status of loneliness research. Thus, the claim is sometimes made that loneliness is as important for health as smoking (Pantell et al., 2013), even though this is not endorsed by direct comparisons (Batty et al., 2021), and is often based on the ‘Table 2 fallacy’ in multivariable analysis (Westreich & Greenland, 2013). There is no need to overstate the crucial role played by loneliness in modern life by making such comparisons. However, research in this field is complicated by the association between the subjective experience of loneliness and more objective aspects of relationships such as social isolation. Unfortunately, too few studies include measures of both loneliness and isolation, but there is some evidence that isolation may be more critical than loneliness for physical health outcomes, while loneliness is implicated in mental ill‐health (Holt‐Lunstad & Steptoe, 2022). This issue is not just of academic interest, because it is relevant to the types of intervention that may be most fruitful in the population. There are certainly wide variations in the magnitude of associations between loneliness and health across studies, and contributions to this Special Issue throw light on this question. Notably, the longitudinal analyses of the German Ageing Study by (Beller, 2023) show that positive affect buffers to adverse association of loneliness with mortality. Thus, the presence of high positive affect greatly diminished the heightened mortality risk of loneliness. This is an interesting finding that helps to account for some of the differences in the outcomes of longitudinal studies and suggests new avenues for addressing the loneliness problem.

Understanding whether loneliness is related not only to mortality but also to negative health trajectories among people with existing illness is important. One difficulty in interpreting these patterns is the overlap of loneliness with depression. Loneliness and depression are closely intertwined, with longitudinal evidence that loneliness predicts future depression over many years, even when genetic risk is taken into account (Lee et al., 2021). The problem is that comorbid depression leads to poorer outcomes among people with a range of serious health conditions including coronary heart disease (CHD), chronic obstructive pulmonary disease and diabetes (Steptoe, 2018). Demonstrating that loneliness impacts on prognosis independently of depression is challenging. One relevant mechanism may be poor adherence to medical advice. The study from Fan et al. (2023) of people with CHD in Singapore shows that the relationship between loneliness and poor quality of life is mediated by low adherence. It is likely that lonely individuals are not supported by encouragement from people around them to follow advice and take medication, and this has negative consequences.

The social context is of course critical to loneliness, as is illustrated by several articles in this Special Issue. One example is the intriguing study of social networking among Chinese students (Sun et al., 2023). This report combined observational with experimental methods to provide convincing evidence that feedback on social networking posts (‘likes’ and comments) are related to lower loneliness and a greater sense of social connectedness. There are certainly dangers in social media activity as well, in that negative comments and feedback may have adverse effects on mental health (Lippke et al., 2021). But the study is a timely reminder that remote communication can have positive consequences, and this is particularly important in the context of the COVID‐19 pandemic.

## LONELINESS DURING THE COVID‐19 PANDEMIC

The COVID‐19 pandemic led to marked increases in psychological distress, depression and anxiety in the population (Covid‐ Mental Disorders Collaborators, 2021). These reactions were elicited partly by fear of infection and worry that the virus might lead to serious illness or even death. But emotional responses were also driven by the impact of regulations put in place to reduce infection. These included enforced isolation; stay at home orders that led to separation from support groups; concerns about employment, education and financial stability, together with reduced access to welfare and health services; and even difficulties in obtaining needed supplies. There was widespread speculation that the pandemic would lead to large increases in loneliness as a result of these experiences. However, this has not materialised in the population in general. A meta‐analysis of 34 studies collecting data from before to during the pandemic documented quite small increases in loneliness (Ernst et al., 2022). A large study involving weekly assessments of loneliness over the first months of the pandemic in more than 38,000 respondents showed that most people maintained low loneliness levels (Bu et al., 2020). In a nationally representative sample of older adults in England, analyses of trajectories from 2018 to the early months of 2020 found some increases in loneliness, but these were much smaller than the rises in depression and poor quality of life (Zaninotto et al., 2022). One explanation may be that the experience of loneliness during the pandemic was rather different from loneliness in general. Loneliness in its traditional sense involves perceptions of a lack of companionship and a feeling that social connections do not match the levels desired by the person. During the pandemic, people did not lack companionship but rather were not able to meet others in person as much as they might have liked.

The strongest evidence for the effects of the COVID‐19 pandemic on loneliness and other emotional states comes from longitudinal studies that could compare outcomes before and after the onset of the crisis. But the study by Jiang and Lee (2023) offers an interesting alternative design, comparing individuals returning to China from overseas who were quarantined for 14 days with nonquarantined people. Daily diaries confirmed that the quarantined group had lower positive affect over this period, although negative affect did not differ. These patterns were moderated by participants' beliefs about the controllability of their emotions, highlighting potentially important individual differences.

Despite the modest increase in loneliness during the pandemic in the population at large, there are marked variations in responses. Young adults, women, people with lower incomes, disability and pre‐existing mental health issues appear particularly at risk for increased loneliness (Fancourt et al., 2021). The studies of loneliness in this Special Issue add to our understanding by focusing on vulnerable and marginalised groups including LGBT persons and individuals with serious health problems (Herrmann et al., 2023; Keller et al., 2023; Lamont et al., 2023). Many LGBT people struggled with loneliness even before the pandemic, but the closure of venues in which they could meet others with a similar identity may have exacerbated loneliness and depression (Herrmann et al., 2023). The analysis of the experience of patients in a psychosomatic clinic undergoing rehabilitation was carried out later in the COVID‐19 pandemic than other studies but showed that loneliness and anxiety mediated links between Covid distress and depression (Keller et al., 2023).

Loneliness was alleviated by social connections for many people during the pandemic. Lamont et al.'s (2023) interesting study of stroke survivors demonstrated how support groups could be maintained remotely using telephone calls and internet contact. Poor health and living alone were associated with poor outcomes, but shared social identity and the sense of being supported played a positive role. But other work has shown that social contact had a negative side for some during the pandemic, with the enforced close contact with household members during periods of lockdown being stressful, and leading to increased domestic abuse (Office for National Statistics, 2020). The detailed examination of the daily experience of Canadian adults in the first months of the pandemic was designed to explore the interesting issue of the benefits of solitude or having time to one's self. Contrary to expectation, desire for solitude was not related to household size. However, periods of solitude were associated with greater positive affect among individuals who had poor or conflictual relationships with the people they lived (Choi et al., 2023). The sample in this study was highly educated, and it was not clear whether households included children, so extension to more diverse populations would be intriguing.

There were also somewhat unexpected findings concerning leisure activities and mental health during the COVID‐19 pandemic in the study conducted by Takiguchi et al. (2023). This study showed that Japanese adults reported a large decrease in leisure activities after the onset of the pandemic but that there was no association between reductions in leisure activity and greater depression. The finding contradicts other longitudinal research in the United States and the UK (Bone et al., 2022; Bu et al., 2022). One explanation may be that the focus of the analysis was on the number of leisure activities rather than type of activity; physical restrictions led to a reduction in leisure activities outside the home, whereas other research has been centred on hobbies and engagement with arts and other activities that can be pursued within the home.

People's ability to engage in outdoor or community activities was reduced in the early months of the pandemic, but levels of restriction varied considerably across countries. One tool that will be valuable in future cross‐national research on the psychological impact of the pandemic is the COVID‐19 Stringency Index (Hale et al., 2021). This attempts to measure the extent of restrictions on movement and activities in multiple countries at different periods of the pandemic. It details large international variations in policy; some countries had few restrictions that were rapidly withdrawn after the first few months, while others such as China have had more enduring constraints on everyday life. It has been shown that more stringent policies were associated with poorer mental health across 15 countries (Aknin et al., 2022), and mapping these variations in relation to loneliness scores over time and across countries would be a fascinating exercise. It would through light on the ways in which public policy impacts loneliness and perceptions of isolation.

## INTERVENTIONS TO REDUCE LONELINESS

A key goal of research into loneliness and health is to formulate interventions that will reduce loneliness, enhance social connectedness and improve mental and physical health outcomes. There are many different approaches to intervention, ranging from in‐depth individual or group CBT to large‐scale population‐based initiatives to encourage interaction (Cacioppo et al., 2015). Numerous intervention methods are being explored. For example, the EU Science Hub has developed an online repository of loneliness interventions in the EU‐27 states that currently lists more than 320 projects delivered by 290 organisations (https://knowledge4policy.ec.europa.eu/composite-indicators/mapping-loneliness-interventions_en). Prominent among these are programmes for connecting people, group/social activities, social prescribing and awareness raising. Published studies have been extensively reviewed, but the results have often been disappointing, with the scientific quality of work being very mixed (Eccles & Qualter, 2021; Gardiner et al., 2018; Hajek et al., 2021). One important issue is how interventions are developed; as Scholz (2023) emphasises in her commentary, it is crucial that interventions are theory‐based and not created on an ad hoc basis.

This Special Issue contributes to the debate on how to intervene in several important ways. The meta‐analysis reported by Li et al. (2023) is a timely review of loneliness interventions for older people in China. Although the effect size was large, the authors note a strong risk of bias that may overexaggerate benefits and plead for higher‐quality research. Nevertheless, the review does suggest that psychological interventions such as CBT and counselling may be valuable, particularly when administered on a group basis. Groups are also crucial to the report about the experience of small parties of young people undergoing a strenuous 2‐day outdoor programme in remote country (Cohen et al., 2023). The study did not include a comparison condition and only examined short‐term effects, but it is notable that the increases in well‐being were as much due to social bonding as to completion of the activity itself. Links between exposure to the natural world, including both green (countryside) and blue (sea, inland waters) spaces, and better mental health have been identified in many countries around the world (White et al., 2021), and understanding relationships with social isolation and loneliness is a fruitful avenue for future research (Leavell et al., 2019).

Another intriguing short‐term study that documents preliminary results focused on the scheme developed by the US branch of the Heartfulness Institute, an international movement promoting meditation and relaxation exercise (Iyer et al., 2023). The 4‐week programme led to greater reductions in loneliness than a waiting‐list comparison, and drop‐out rates were low. Other research on this programme has reported favourable changes in measures of psychological well‐being, so effects were not limited to loneliness. A key challenge is to sustain changes after the formal intervention period ends. When people are no longer part of a study, the support from the research or therapeutic team that they enjoyed is cut off, and enthusiasm for continuing with activities or exercises may fade. However, it is possible that some of the issues addressed in other articles, such as social identity (Lamont et al., 2023), positive affect (Beller, 2023) and positive feedback (Sun et al., 2023), could be applied in intervention studies and help maintain change in the long term.

Taken together, the studies detailed in this Special Issue conform the vitality of applied psychological research on loneliness and health. The field is flourishing in many parts of the word, using diverse methodologies, study samples and approaches. Bringing these activities together provides a showcase that furnishes many opportunities for future research. Readers will find that these articles stimulate ideas about new directions for studies that help us better understand and reduce loneliness.
